# The hidden health equity crisis: readability assessment of online information regarding periodontitis-diabetes relationship

**DOI:** 10.3389/fpubh.2025.1710193

**Published:** 2025-12-17

**Authors:** Eugene Gamble, Peter Chami, Heather Harewood, Tamara Nancoo

**Affiliations:** The University of the West Indies, Bridgetown, Barbados

**Keywords:** bidirectional relationship, diabetes, digital health, health literacy, health equity, online health information, patient education, periodontitis

## Abstract

**Background:**

Despite strong evidence linking periodontitis as a risk factor for diabetes, this association remains significantly underrepresented in online health information. Given that online resources serve as a primary source of health education, this study examines the content coverage and readability of diabetes-related websites from government and health organizations.

**Methods:**

A total of 154 health and diabetes-related websites were screened, with only 28 containing any reference to periodontitis-diabetes interrelationship. Readability analysis was specifically conducted on this subset of 28 sites using Flesch–Kincaid Grade Level (FKGL), Gunning Fog Index (GFI), Simple Measure of Gobbledygook (SMOG), Coleman–Liau Index (CLI), and Flesch Reading Ease (FRE) Score. Readability scores were then compared to the American Medical Association's recommended fifth-to-sixth-grade reading level (~10–12 years old).

**Results:**

Only 15 websites (9.7%) explicitly identified periodontitis as a risk factor for diabetes, highlighting a major gap in online health education. Readability analysis of sites that cover any part of the periodontitis-diabetes interrelationship (n = 28) showed that most content was written at a level too complex for general audiences. FKGL (mean = 12.0, p < 0.001), GFI (mean = 12.0, p < 0.001), SMOG scores (mean = 13.6, p < 0.001), CLI (mean = 13.6, p < 0.001), and FRE scores (mean = 43.9, p < 0.001) indicate that much of the material requires college-level reading proficiency (typically 18+ years old).

**Conclusions:**

The overwhelming majority of online diabetes resources fail to acknowledge periodontitis as a risk factor for diabetes, despite well-established evidence supporting this link. Additionally, the readability of available materials is significantly above recommended levels for public comprehension. This accessibility gap may lead to a lack of awareness among patients and healthcare professionals, further contributing to health inequality. To enhance health literacy and encourage integrated care approaches, websites should prioritize both simplifying their content and ensuring the periodontitis-diabetes relationship is clearly communicated.

## Introduction

Diabetes Mellitus (DM) and periodontitis are two highly prevalent Non-communicable diseases (NCDs), affecting ~830 million and more than 1 billion people worldwide, respectively (1, 2). These conditions have well-established bidirectional links. Recent epidemiological evidence indicates that individuals with periodontitis are at significantly increased risk of developing DM (adjusted hazard ratios: 1.19–1.33) (3), highlighting the need for integrated management strategies.

Research has identified several biological mechanisms, including cytokine-mediated mechanisms (4), oxidative stress (5), and immune dysregulation (6), believed to explain how periodontitis contributes to poor glycemic control and increases DM-related complications such as retinopathy and nephropathy (3, 7–9). Conversely, DM is a significant risk factor for the development and progression of periodontitis (10, 11). Despite growing evidence supporting this connection, awareness remains limited among non-dental healthcare professionals and people living with diabetes (PLWD) (12, 13). Integrating periodontal health into DM education and management strategies is therefore critical for improving patient outcomes (7).

In the digital age, many patients rely on online sources for health information even before consulting health care providers (14, 15). The accuracy, and readability of these resources are essential for effective patient education. However, disparities in health literacy can pose a significant challenge. Individuals with lower socioeconomic status (SES) are more likely to have limited health literacy (16), which is associated with poorer health outcomes and increased healthcare costs (17). Given that many PLWD belong to socioeconomically disadvantaged groups (18), ensuring that digital resources are clear, evidence-based, and easily understandable is crucial for bridging health disparities and empowering patients to manage their condition effectively (19). Numerous studies have assessed the readability of online health information, consistently finding that it is often written at levels too advanced for the general public. Systematic reviews and meta-analyses indicate that materials across various health topics typically require a 10th to 15th grade reading level, far exceeding the recommended 5th to sixth grade standard (20, 21). This issue is particularly evident in diabetes education materials, where online resources on topics like cardiovascular complications or general management have been shown to have poor readability, understandability, and actionability, potentially limiting their utility for patients with lower health literacy (22). Despite these findings, gaps persist in evaluating specific interrelationships, such as between periodontitis and diabetes.

DM associations and health organizations play a key role in providing online health education (23). However, it remains unclear to what extent these sources address the periodontitis-DM link, offer actionable recommendations, or present information in an understandable format.

This study systematically evaluated English-language online health resources from government, DM, and medical websites worldwide. We assessed content coverage regarding whether these sources acknowledge the bidirectional relationship between periodontitis and DM. This includes recognition that periodontitis can be both a complication of and a risk factor for DM, and that DM similarly contributes to the development and progression of periodontitis, whether they offer treatment recommendations, cite scientific references, and indicate the currency of the content.

Furthermore, for the subset of resources that included any information on this relationship, we evaluated the readability of the relevant content using five validated readability metrics, including the Simple Measure of Gobbledygook (SMOG) (24), Flesch Reading Ease (FRE) (25), the Gunning Fog Index (GFI) (26), Flesch-Kincaid Grade Level (FKGL) (27), and the Coleman–Liau Index (CLI) (28). These tools have been used to determine whether health information is appropriately written for the general public (29, 30).

By identifying gaps in content coverage and readability, this study seeks to inform strategies that enhance the dissemination of evidence-based information on the periodontitis-DM link and to empower PLWD to make informed health decision.

## Method

### Design

This study employed a cross-sectional descriptive research design using a structured content analysis of publicly available websites. The objective was to evaluate content coverage across all included websites and the readability of online health information specifically for those resources that included details on the bidirectional relationship between periodontitis and DM across a diverse set of health-related websites. Data collection and analysis were conducted using a predefined protocol and binary coding framework.

### Definition of key terms

For consistency and clarity, the following operational definitions were used in this study.

- **Periodontitis**: a chronic inflammatory disease affecting the supporting structures of the teeth, characterized by progressive attachment loss and bone destruction. In this study, any website reference to “periodontitis,” “gum disease,” or “periodontal disease” was considered as relevant content.
- **Diabetes mellitus (DM)**: a metabolic disorder characterized by chronic hyperglycemia. In this study, both type 1 and type 2 diabetes were included under the term “diabetes” unless specified otherwise by the website.
- **Readability**: the ease with which written content can be read and understood by a target audience. In this study, readability was quantitatively assessed using standardized tools (e.g., Flesch Reading Ease, SMOG Index, etc.).
- **Presence of content**: a binary coding (Yes/No) indicating whether specific predefined information (e.g., acknowledgment of the periodontitis-DM link, treatment guidance) was found on a webpage.

### Website selection

To assess the availability of information on the bidirectional relationship between periodontitis and DM, we employed a structured, multistep approach to identify relevant online resources. The process began with manual searches of authoritative sources, including recognized diabetes associations (e.g. https://diabetes.org/) and national health ministries (e.g. https://www.nhs.uk/). These manually sourced URLs served as a foundational set of high-priority websites known to be relevant to the study's scope. To expand and diversify this initial pool, we subsequently queried three artificial intelligence (AI) engines (ChatGPT 4.0, Copilot, and Gemini) using the prompt “*List official websites of Diabetes associations and government health agencies that provide educational content or public health guidance*”. This multi-AI engine approach was chosen to leverage the diverse knowledge bases and algorithms of each platform, aiming to uncover additional resources that might not surface through manual search alone. While AI-generated responses can vary over time due to model updates, using multiple engines helped reduce reliance on any single source.

To further guide the AI engines and enhance the relevance of their outputs, the manually identified websites were also provided as examples during the querying process. This hybrid strategy was designed to ensure both depth and breadth in identifying educational and public health resources. After combining the lists, we removed duplicates and applied the following inclusion/exclusion criteria.

**Inclusion criteria:**

- **Official websites:** the website had to be the official website of a recognized diabetes association, government health agency, or global health organization.
- **English language:** website content had to be in English.

**Exclusion criteria:**

- **Non-english websites:** Websites whose language was not English.
- **Non-health organization websites:** Websites of commercial entities (e.g., pharmaceutical companies, medical device manufacturers), individual healthcare practices, news outlets, personal blogs, or other non-official health organizations.
- **Fabricated or inactive links:** websites that resulted in HTTP errors or were no longer active.

**Figure 1:**
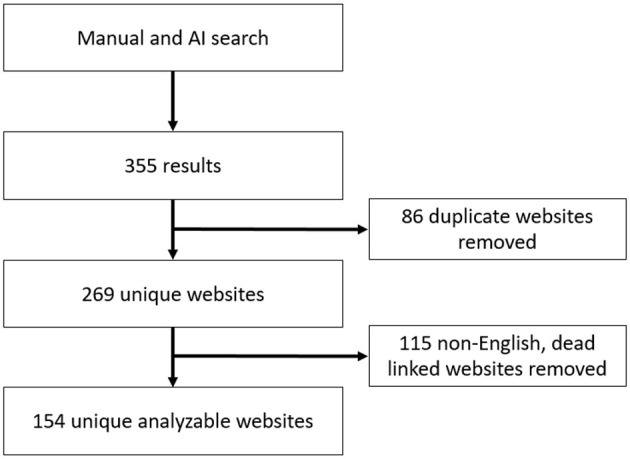
Website selection flowchart.

This selection process ensured that only high-quality, relevant online resources were included for analysis (Figure 1) and are included in Supplementary Table S1 for transparency and reproducibility. All website identification and content searches, including AI queries and Google site specific searches, were conducted during the first quarter of 2025.

A systematic search protocol was developed to ensure consistency and reproducibility across all websites. Each of the listed websites were searched for literature and resources on the relationship between periodontitis and DM. Sites were searched individually using Google, the world's most widely used search engine (31), and its site-specific search functions (site: www.website.com) combined with the Boolean search terms “gum” OR “gums” OR “periodontitis” OR “periodontal” AND “diabetes”. These terms were selected to capture a wide range of references to periodontal disease and to ensure comprehensive coverage of relevant content.

### Analysis

Search results were cataloged, and each relevant webpage was reviewed using a standardized binary assessment tool specifically developed for this study. The tool was designed to record the presence (“Yes”) or absence (“No”) of information around the study's core research questions. The binary (Yes/No) format was selected to simplify analysis and reduce subjectivity in evaluating online health content. It included five predefined criteria:

- Acknowledgment of periodontitis as a risk factor for DM
- Presence of treatment recommendations
- Acknowledgment of DM as a risk factor for periodontitis
- Presence of references or citations
- Age of the resource (if available)

To enhance validity, the tool was pilot-tested on a random sample of 10 websites by two reviewers. Minor refinements were made for clarity and consistency. Following this, all websites were assessed by one primary reviewer, with a random sample (*n* = 31, 20.1%) independently reviewed by a second researcher for discrepancies and further discussion if required. Agreement between reviewers was quantified using Cohen's Kappa, which yielded a value of 0.81, indicating almost perfect agreement.

To prepare text for readability analysis, the relevant sections discussing the periodontitis-DM relationship were extracted and copied as plain text. HTML formatting, hyperlinks, and embedded media were removed. Irrelevant elements such as navigation menus, footnotes, tables, charts and images were also removed. Orthographic elements such as colons, semicolons, and decimal points within sentences were removed as they could artificially affect sentence length detection.

Readability analysis was conducted specifically on the relevant sections of text from the 28 websites that contained any information on periodontitis-DM interrelationship, using multiple validated readability metrics calculated via Infyways readability checker (32), an online tool that was manually validated to ensure legitimacy and accuracy of outputs. These included:

- Flesch Reading Ease (FRE) Score
- Simple Measure of Gobbledygook (SMOG) Index
- Gunning Fog Index (GFI)
- Flesch–Kincaid Grade Level (FKGL)
- Coleman–Liau Index (CLI)

These five metrics were selected for their widespread validation and use in health communication research, as they provide complementary assessments of text complexity, with some emphasizing syllable counts for semantic difficulty, and others focusing on character or sentence structure for syntactic ease, resulting in a more robust evaluation than any single formula (23–28). This multi-metric approach mitigates limitations of individual tools. Specifically: FRE score: calculates readability on a 100-point scale (higher scores indicate easier text) using average sentence length (ASL) and average syllables per word (ASW), with the formula: Score = 206.835 – (1.015 × ASL) – (84.6 × ASW).

SMOG index: estimates the US grade level required using the number of polysyllabic words (3+ syllables) and number of sentences, with the formula: Grade = 1.043 x √(polysyllables × (30/Sentences)) + 3.1291. It is particularly suited for health texts due to its focus on complex vocabulary.

GFI: estimates US grade level based on ASL and the percentage of complex words (3+ syllables, excluding familiar jargon), with the formula: Index = 0.4 x (ASL + percentage of complex words).

FKGL: provides US grade level also using ASL and ASW, with the formula: Grade = (0.39 × ASL) + 11.8 × ASW – 15.59. It adapts the FRE for educational grading.

CLI: Estimates US grade level using average characters per word and average words per sentence, with the formula: Grade = (5.89 × characters per word) + (0.3 × sentences per 100 words) – 15.8. It is advantageous for automated analysis as it avoids syllable counting.

These five readability tests assess readability based on word difficulty and sentence length, each applying different weighting factors. Their complementary strengths provide a more comprehensive and robust assessment of how easily online health information can be understood by the general public. Readability scores are rough estimates and should be interpreted with caution. Results are presented rounded to one decimal place to avoid implying false precision.

The online tool was chosen as the calculation method due to its support for all five selected formulas in a singles, free, and user-friendly platform, ensuring consistent application across metrics. To address known variability in online calculators (33), we compared it's results against readable.com using standard sample texts, confirming low grade variability (±0.5 grades).

These indices were selected because they are among the most widely used and validated readability tools in health communication research, collectively capturing both syntactic and semantic complexity. Their complementary strengths provide a more comprehensive and robust assessment of how easily online health information can be understood by the general public.

The FRE is a 100-point scale, with higher scores indicating easier-to-read text. Readability scores that yield U.S. school grade levels (FKGL, GFI, SMOG, and CfLI) indicate the number of years of education typically required to understand the content. For context, a sixth-grade level corresponds to the reading ability of an average 11–12-year-old (upper elementary school). Scores from 13 to 16 reflect college-level readability, while scores above 16 suggest postgraduate-level complexity (24–28). Normality of the readability score distributions was assessed using the Shapiro-Wilk test. All scores were approximately normally distributed. Readability scores, including mean, median, standard deviation (SD), and range were subsequently calculated. This quantitative analysis provided insights into the extent to which organizations address the relationship between these two NCDs. All statistical analyses were conducted using R version 4.5.0 (2025-02-02).

The American Medical Association (AMA) and National Institutes of Health (NIH) recommend that patient-directed health information be written at or below the fifth- to sixth-grade level to ensure user-friendliness for the general public (34). To assess whether readability scores significantly exceeded the recommended 5.5-grade level, one-sample *t*-tests were performed for each index that uses this scale. Statistical significance was set a *p* < 0.05. Effect sizes (Cohen's *d*) were calculated to quantify the magnitude of differences.

## Results

### Website selection outcomes

The initial search yielded a combined list of 355 websites from manual identification and AI-generated queries. After removing duplicates and applying inclusion/exclusion criteria, 154 websites were selected for analysis (Figure 1). These included official sites from diabetes associations, government health agencies, and global health organizations, as detailed in Supplementary Table S1.

### Prevalence of information on the DM-periodontitis links

Of the 154 websites analyzed, only 15 (9.7%) recognized periodontitis as a risk factor for DM while 28 (18.2%) acknowledged the reverse fact that DM increases the risk of developing periodontitis. The remaining 126 websites (81.8%) did not address the DM-periodontitis interaction at all.

### Presence of treatment recommendations

Among the 154 websites, 21 (13.6%) included treatment recommendations for managing periodontitis in individuals with DM. These recommendations varied in specificity, with some sites providing general advice on maintaining oral hygiene and others offering more detailed guidance on professional periodontal treatment and its impact on DM management.

### Readability analysis of website content

The assessed text from the sites had an average word count of 352 words (median = 319, SD = 197, range = 79–857). The readability of content across the analyzed webpages varied considerably.

- Flesch–Kincaid grade level (FKGL)

FKGL scores ranged from 5.6 (upper elementary level) to 16.6 (college level), with a mean of 12.0 (Table 1). A *t*-test confirmed that FKGL scores were significantly higher than the AMA-recommended fifth- to sixth-grade level [*t*(27) = 13.3, *p* < 0.001], with a large effect size (Cohen's *d* = 2.52). Moreover, 96.4% of websites had FKGL scores exceeding the sixth-grade level.

- Gunning fog index (GFI)

**Table 1:** Descriptive statistics of readability scores and word count for analyzed websites.

**Statistic**	**FKGL**	**GFI**	**SMOG**	**CLI**	**FRE**	**Word count**
Mean	12.0	12.0	13.6	13.6	43.9	352
Median	12.0	12.2	13.8	13.6	42.8	319
SD	2.6	2.5	2.0	2.6	14.7	197
Range	5.6–16.6	6.6–17.2	7.3–17.3	7.7–19.4	18.9–81.7	79–857

**Figure 2:**
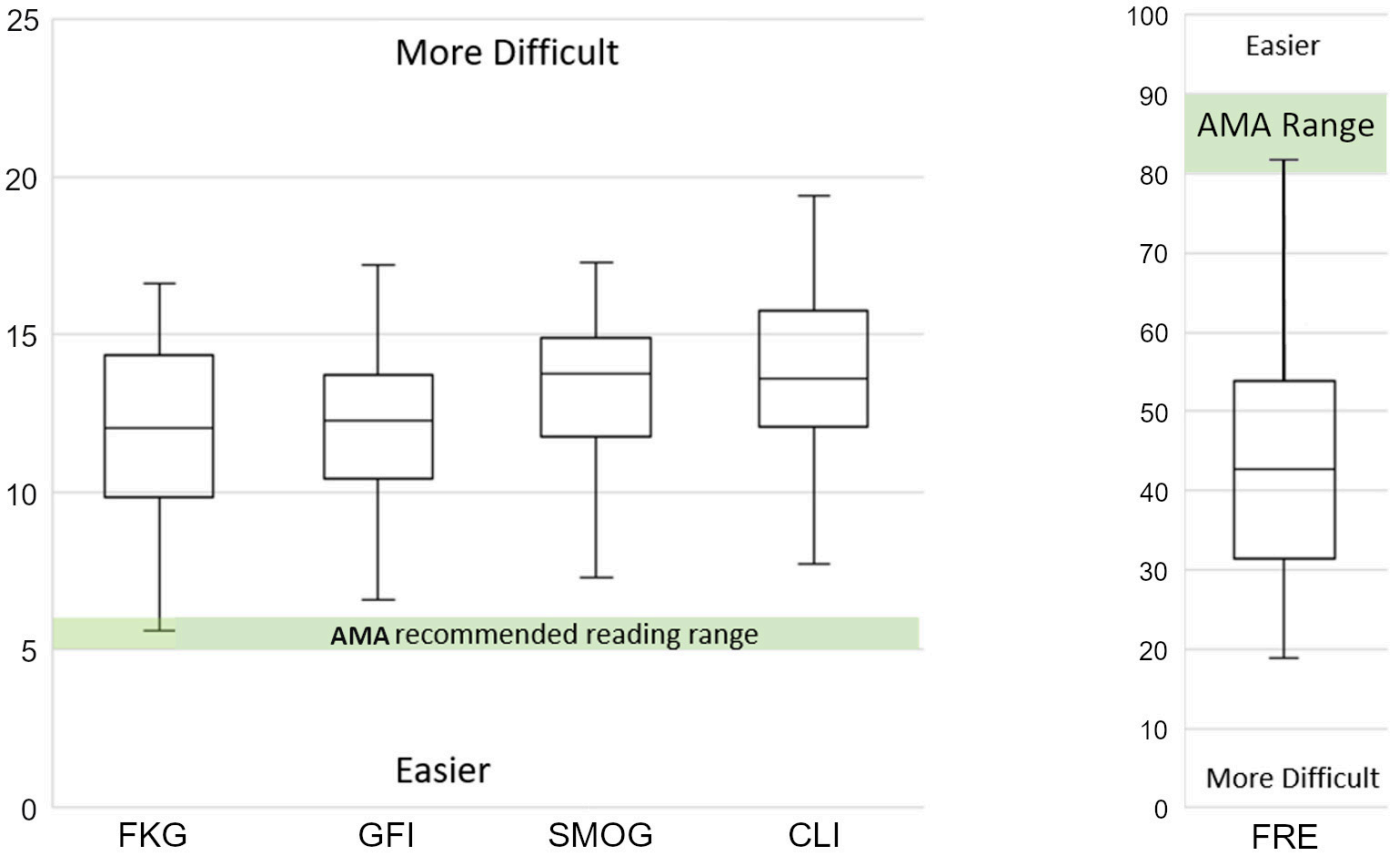
Boxplot of results with American Medical Association (AMA) recommended reading range highlighted (Green Area). FKG, Flesch–Kincaid grade level, GFi, gunning fog index, SMOG, simple measure of gobbledygook; CLI, Coleman–Liau index), and FRE, Flesch reading ease.

The GFI ranged from 6.6 (~7th-grade level) to 17.2 (postgraduate level) (Table 1). The mean GFI score of 12.0 (12th-grade level) was also significantly above the AMA-recommended level (Figure 2) [*t*(27) = 14.0, *p* < 0.001], with a large effect size (*d* = 2.65), and 100% of websites exceeded the sixth-grade threshold.

- Simple measure of gobbledygook (SMOG) index

The SMOG Index, had a wide range (7.3–17.3) with a mean of 13.6 (college level) (Table 1). Statistical testing confirmed SMOG scores to be significantly above the recommended readability level (Figure 2) [*t*(27) = 20.4, *p* < 0.001], yielding a very large effect size (*d* = 3.85). Notably, 100% of the websites had SMOG scores above the sixth-grade level.

- Coleman–Liau index (CLI)

The CLI, had a range of 7.7 to 19.4 (8th-grade to graduate level), with a mean of 13.6 (college level) (Table 1). CLI scores were significantly above the recommended level (Figure 2) [*t*(27) = 16.8, *p* < 0.001), with a very large effect size (*d* = 3.17), and 100% of websites exceeded the sixth-grade level.

- Flesch reading ease (FRE) score

FRE scores, where higher scores indicate easier-to-read text, ranged from 18.9 (college graduate level) to 81.7 (6th-grade level), with a mean of 43.9 (11th grade level) and a median of 42.8 (Table 1). To allow statistical comparison the grade 5.5 benchmark was converted to an equivalent FRE score of 85. A one-sample *t*-test showed that the observed mean FRE was significantly lower (i.e. more difficult) than the benchmark [*t*(27) = −14.8, *p* < 0.001), with a very large effect size (*d* = 2.8). These results indicate that the sampled websites are substantially more difficult to read than the recommended level (Figure 2). Notably, several websites had FRE scores below 30, suggesting that their content may be too complex for a general audience.

These metrics do not always align perfectly, as each uses different algorithms and language features to determine readability. To address these discrepancies, we reported the full range and central tendencies (mean, median, and standard deviation) of each readability score and interpreted the results in aggregate. This approach enabled us to identify consistent trends across tools, even when absolute values differed.

The 95% confidence intervals for the mean readability scores (e.g., FKGL: 11.0–13.1, GFI: 11.1–13.0, SMOG: 12.7–14.4, CLI: 12.6–14.6, FRE: 38.2-49.6) (Figure 2).

### References and citations

Scientific citations were present in 15 of the 28 websites containing relevant information (53.6%), suggesting that more than half of these sources referenced peer-reviewed literature or other authoritative resources to support their claims. The remaining 13 websites (46.4%) did not provide scientific references, potentially limiting the credibility and reliability of the information presented.

### Age of content

Among the 28 websites, publication dates were identifiable for 16 sites (57.1%). Of these:

- 12 websites (75% of those with identifiable dates) published their information from 2020 or later, indicating relatively recent content.
- 4 websites (25%) published their information before 2020.

While the presence of older publication dates does not automatically equate to a lack of credibility, it raises concerns about the currency of the information presented. Medical knowledge and guidelines evolve over time, particularly in areas like DM management and the understanding of its links to other conditions. Therefore, information published before 2020 may not reflect the most up-to-date research or clinical recommendations. The credibility of these older websites would depend on several factors that were not systematically assessed in this study, such as whether the content has been reviewed or updated since its initial publication, the reputation of the publishing organization, and the alignment of the information with current evidence-based practices. Without explicit information on review or update dates, the reliability of the recommendations on these older sites should be interpreted with caution.

The remaining 12 websites (42.9%) did not provide any information on publication dates, limiting the ability to assess the timeliness of the content for these sources. The age of the information is crucial for ensuring that the recommendations and data are up-to-date and reflective of current research findings and clinical practices.

## Discussion

### Limited recognition and public health implications

The findings of this study reveal a significant gap in the availability of online health resources addressing the bidirectional relationship between DM and periodontitis. This indicates a lack of information on the links and a notable disparity in how the bidirectional relationship between the two conditions is communicated, highlighting a major gap in online health education. A gap with substantial public health implications. As shown in the results, < 10% of websites acknowledged periodontitis as a risk factor for DM, and fewer than 20% recognized DM as a risk factor for periodontitis. These findings highlight a significant barrier to public understanding, as much of the material is written at a high school or college level, underscoring the urgent need for revising health communication strategies. This lack of understandable, well-integrated information represents a missed opportunity to improve health outcomes, such as glycemic control, through clear, actionable guidance. This is concerning given that the association between these conditions has been well established by the European Federation for Periodontology for decades (3).

Many patients rely on Internet resources for health information (15), yet only a small percentage of websites provide treatment recommendations to the co-occurrence of these conditions. When major DM organizations and government health websites overlook this link, patients may fail to recognize periodontal care as a key component of DM management.

A contributing factor may be that much of the research on the periodontal-DM link is published in dental journals rather than in medical or public health literature. As a result, the dissemination of this information to physicians, public health professionals, and PLWD remains limited. This reflects the broader issue of research silos, where valuable health findings fail to cross disciplinary boundaries, limiting their translation into public health policies and clinical practice.

This lack of emphasis may reflect broader gaps in clinical practice guidelines. While some DM guidelines acknowledge oral health, they often fail to highlight its bidirectional impact on DM outcomes. Therefore, there is a pressing need for more prominent and integrated inclusion in the DM-periodontitis relationship within clinical guidelines for health care providers. Public health initiatives should also prioritize integrating oral health in national DM programs.

To bridge these gaps, greater integration of periodontal research into medical and public health literature is advisable, alongside stronger collaboration between dental and medical researchers. Medical and public health journals could actively solicit and prioritize research examining systemic impacts of periodontal disease, including its bidirectional relationship with DM. This could involve dedicated special issues or thematic sections focused on oral-systemic links. Additionally, joint conferences and interdisciplinary workshops could foster collaborations, leading to shared research projects and the development of integrated clinical guidelines. Finally, funding agencies could prioritize research grants that encourage collaborative efforts between dental and medical research teams to investigate the underlying mechanisms and clinical implications of the periodontitis-DM nexus.

### Readability, AI and health equity

The knowledge gap hypothesis suggests that individuals with higher socioeconomic status and greater access to education are more likely to acquire and apply new health information, while those from disadvantaged backgrounds may be left behind, exacerbating health disparities (35). PLWD often belong to lower socio-economic groups, which are also more likely to have lower levels of formal education (16, 18). Given these demographic challenges, it is critical that health information be presented in a way that is understandable to this population. As demonstrated by our readability analysis in the results, the online information available often exceeds the recommended reading levels for patient education materials. The 95% confidence intervals for the mean readability scores confirm that even the lower bounds of the metrics remain well above the recommended level (or in the case of FRE, well below), underscoring the urgent need for revising health communication strategies. Complex medical terminology and high readability demands can create barriers to understanding, preventing those with the greatest need from benefiting from the information.

The current lack of widely available, easy-to-understand information on the periodontitis-DM link may further reinforce this divide, limiting awareness and preventive action among those most at risk. Ensuring that online health resources are written in plain language, with clear recommendations, is essential to addressing this gap. This is a matter of health equity and can empower marginalized populations to take proactive steps in managing their health.

Advances in AI offer promising solutions for enhancing the readability of patient education materials. AI-powered language models can help simplify complex medical terminology, adjust readability levels, and tailor content to diverse literacy needs. Automated tools such as natural language processing systems can assess text complexity and generate clearer, more comprehensible versions of health information.

Integrating these technologies into the development of online health resources could help bridge communication gaps and ensure that essential information about the periodontitis-DM link is both understandable and actionable for all patients, particularly those from disadvantaged backgrounds. This, however, should be approached with caution as AI may introduce inaccuracies or oversimplify a topic and human oversight should always accompany such use.

Future studies could enhance assessments by incorporating tools like the Patient Education Materials Assessment Tool (PEMAT), developed to assess understandability and actionability of patient education material, which goes beyond text readability metrics (36). This would provide a more holistic view of online resource effectiveness.

## Conclusion

By analyzing these results, we identify significant gaps in the online dissemination of information regarding the periodontitis-DM relationship. Not only is the information scarce, but when it is available, it is often presented in a manner that may be too complex for the general public to easily understand. This issue of readability is particularly critical when considering patients from lower socioeconomic backgrounds, who are disproportionately affected by both DM and periodontitis and often have lower levels of health literacy. The use of complex language and high reading levels in online resources creates a significant barrier for these deprived patients, hindering their ability to access and comprehend essential information about managing their conditions and understanding the crucial bidirectional link between them. This lack of accessible information can exacerbate existing health disparities, preventing those most in need from taking proactive steps to improve their health outcomes. This emphasizes the need for more comprehensive and understandable resources to support patients in understanding the critical links between periodontitis and DM.

### Recommendations

Based on the findings of this study, several actionable recommendations are proposed to improve the comprehensibility and dissemination of information on the relationship between periodontitis and DM across official health communication platforms:

1. **Integrate periodontal-DM messaging into public health communications** Health agencies and DM associations should explicitly incorporate information about the bidirectional link between periodontitis and DM into patient-facing materials, including webpages, downloadable brochures, and health campaign content. This inclusion is essential given the established scientific consensus and the potential for improved health outcomes through interdisciplinary care.
2. **Adopt readability best practices** Organizations should routinely assess and revise their educational materials to meet recommended readability levels, particularly the fifth- to sixth-grade level advised by the American Medical Association. Simple language, shorter sentences, and the use of visual aids can enhance comprehension, especially for populations with limited health literacy.
3. **Establish cross-disciplinary content review panels** Public health institutions and DM organizations should collaborate with dental professionals when developing content related to metabolic and oral health. This interdisciplinary approach can ensure that messaging is accurate, up-to-date, and reflective of emerging scientific evidence.
4. **Update and audit online content regularly** Many websites lacked publication or update dates, making it difficult to assess information currency. Institutions should implement regular audits and prominently display revision dates to improve transparency and encourage trust in the accuracy of their materials.
5. **Provide clear, actionable guidance for patients** Beyond simply acknowledging the association between periodontitis and DM, websites should provide practical advice, such as the importance of regular dental check-ups, oral hygiene strategies, and coordination between dental and medical care providers.
6. **Promote awareness through global health campaigns** Given the global burden of DM and periodontitis, international health organizations such as the WHO should lead or support awareness campaigns that highlight oral-systemic health links, especially in regions with high DM prevalence and low dental care access.

By addressing these gaps in health communication, organizations can support more informed self-management among patients with DM and contribute to improved prevention and control of both conditions.

### Limitations

This study has several limitations that should be considered when interpreting its findings.

The study was limited to English-language websites, which may not reflect the availability of information in non-English-speaking countries. Future research should assess global variations in the dissemination of this information.

Many websites did not provide clear publication dates, making it difficult to assess whether the available information was up to date. Given the evolving nature of medical guidelines, future research should explore how frequently such resources are updated and whether they align with current evidence. In addition, the dynamic nature of web content means that our findings represent a snapshot in time.

While the use of multiple readability formulas provides a quantitative assessment of text complexity, these tools have inherent limitations. They primarily rely on sentence length and word syllables, and do not consider other elements of comprehension, such as visual aids, layout, or actionability (36). They may overestimate readability if complex concepts are explained with diagrams or underestimate it if unavoidable health jargon is used. Even combining formulas does not fully address these gaps, as they remain text-centric and may not reflect real world understanding by various audiences.

Readability formulas are fundamentally rough estimates that do not capture nuances like reader prior knowledge, motivation, or contextual factors, which can sometimes lead to misleading conclusions about how accessible a text truly is. They can also be sensitive to text preparation and calculator variability, as discussed in the methods, and do not evaluate qualitative aspects like clarity of explanations or logical flow. These shortcomings highlight the need for supplementary assessments in future studies to provide a more holistic evaluation of health information effectiveness.

Furthermore, this study did not systematically account for potential confounding factors that could influence the readability scores and the presence of information. For instance, the target audience of a website (e.g., patients vs. healthcare professionals), the funding source (e.g., government, non-profit, commercial), and the specific purpose of the website (e.g., general information, specific product promotion) could all impact the complexity and content of the information presented. Future research should aim to control for or analyse the influence of these and other potential confounding variables to provide a more nuanced understanding of the factors associated with the dissemination and readability of information regarding the periodontitis-DM link.

Additionally, it is important to note that artificial intelligence (AI) engines were utilized in the initial stage of this study to generate a list of potentially relevant websites. While this approach enhanced the efficiency and breadth of the initial search, the inherent limitations and potential biases of the AI algorithms could have influenced the scope of websites identified for subsequent analysis. The final selection of websites for analysis included manually derived sites and manually reviewed with adherence to predefined inclusion and exclusion criteria.

## Data Availability

The original contributions presented in the study are included in the article/Supplementary material, further inquiries can be directed to the corresponding author.
