# Supplementary material for: The hidden health equity crisis: readability assessment of online information regarding periodontitis-diabetes relationship
Source: Front Public Health. 2025 Dec 17;13:1710193. doi: 10.3389/fpubh.2025.1710193 (PMC12753868; doi:10.3389/fpubh.2025.1710193)
Supplement: Supplementary file 1 [file Table_1.docx]

Supplementary Table S1

| www.aace.com |
| --- |
| www.abcd.care |
| www.acbrd.org.au |
| www.sdaihc.org |
| www.aasdonline.com |
| www.bahamas.gov.bs |
| www.dhabarbados.org |
| www.bcdiabetes.ca |
| www.unescwa.org |
| www.belizediabetes.org |
| www.beyondtype1.org |
| www.beyondtype2.org |
| www.cbmr.ku.dk |
| www.cdecb.ca |
| www.childrenwithdiabetes.com |
| www.collegediabetesnetwork.org |
| www.dab-bd.org |
| www.dap.org.pk |
| www.daz.org.zm |
| www.diabeteseducation.ie |
| www.insulin100.eu |
| www.diabetes.bm |
| www.diabetes.ca |
| www.diabetes.co.uk |
| www.diabetes.dk |
| www.diabetes.fi |
| www.diabetes.ie |
| www.srilankadiabetesfederation.lk |
| www.diabetes.no |
| www.diabetes.org |
| www.diabetes.org.cy |
| www.diabetes.org.nz |
| www.diabetes.org.sg |
| www.diabetes.org.uk |
| www.diabetes.se |
| www.diabetesaction.ca |
| www.diabetesaction.org |
| www.echodiabetes.org |
| www.diabetespac.org |
| www.diabetesadvocates.org |
| www.diabetesaustralia.com.au |
| www.diabetesbotswana.org |
| www.diabetesdaily.com |
| www.diabeteseducator.org |
| www.diabeteseducatorscalgary.ca |
| www.diabetesfiji.com |
| www.diabetesvoice.org |
| www.diabetes-hk.org |
| www.diabeteshopefoundation.com |
| www.diabetesindia.com |
| www.diabetesinscotland.org.uk |
| www.nhf.org.jm |
| www.diabetesmalaysia.org.my |
| www.diabetesmalta.org |
| www.apsa.mu |
| www.path.org |
| www.nepaldiabetesassociation.com |
| www.health.hawaii.go |
| www.diabetesnigeria.org |
| www.omandiabetes.org/en |
| www.diabetesphilippines.org |
| www.diabetesresearch.org |
| www.diabetessa.org.za |
| www.diabetesscholars.org |
| www.diabetessisters.org |
| www.diabetesonthenet.com |
| www.diabetessociety.com.au |
| www.health.tas.gov.au |
| www.diabetes-tt.org |
| www.ugandadiabetesassociation.org |
| www.diabetesvic.org.au |
| www.diabeteswa.com.au |
| www.diabeteswellness.net |
| www.diversityindiabetes.org |
| www.dmi.or.ke |
| www.dmthai.org |
| www.dppos.bsc.gwu.edu |
| www.drwf.org.uk |
| www.dyf.org |
| www.easd.org |
| www.eeed.gr |
| www.egyda.org |
| www.emiratesdiabetessociety.org |
| www.endo-metab.ca |
| www.fend.org |
| www.ghanahealthservice.org |
| www.dpi.gov.gy |
| www.iddt.org |
| www.ideg.org |
| www.idf.org |
| www.dnig.ca |
| www.internationaldiabetesnursing.org |
| www.diabetesindia.org.in |
| www.insulin-pumpers.org |
| www.ispad.org |
| www.jdrf.org |
| www.jdrf.org.au |
| www.joslin.org |
| www.kdskuwait.com |
| www.thekdsg.or.ke |
| www.lda.org |
| www.msdiabetes.org |
| www.ndep.nih.gov |
| www.ndss.com.au |
| www.nhs.uk |
| www.nice.org.uk |
| www.niddk.nih.gov |
| www.gracelanddiabetesfoundation.org |
| www.nzssd.org.nz |
| www.padfsg.com |
| www.panafrican-med-journal.com |
| www.qda.org.qa |
| www.racgp.org.au |
| www.sdea.org.sa |
| www.e-district.org/sites/scldef |
| www.t1international.com |
| www.vade.org.vn |
| www.weds-wales.co.uk |
| www.who.int |
| www.integrateddiabetes.com |
| www.worlddiabetesfoundation.org |
| www.woundscanada.ca |
| www.helmsleytrust.org |
| www.lilly.com |
| www.diabetesmine.com |
| www.diatribe.org |
| www.health.gov.au |
| www.bahamas.gov.bs/health |
| www.health.gov.bb |
| www.health.gov.bz |
| www.canada.ca/en/health-canada.html |
| www.moh.gov.gh |
| www.health.gov.gy |
| www.mohfw.gov.in |
| www.hse.ie |
| www.moh.gov.jm |
| www.health.go.ke |
| www.moh.gov.my |
| www.health.govt.nz |
| www.health.gov.ng |
| www.nhsrc.gov.pk |
| www.doh.gov.ph |
| www.moh.gov.sg |
| www.health.gov.za |
| www.health.gov.tt |
| www.health.go.ug |
| www.hhs.gov |
| www.moh.gov.zm |
| www.mohcc.gov.zw |
| www.moh.gov.et |
| www.diabetesfoundationindia.org |
| www.insulinforlife.org |
| www.moh.gov.bt |
| www.diabetesafrica.org |
